# Revealing the U-shaped nonlinear relationship between lipid accumulation product levels and cardiovascular disease risk using the large CHARLS cohort study in China

**DOI:** 10.1186/s12889-025-25096-8

**Published:** 2025-11-14

**Authors:** Yuan-Bo Zhang, Chi Chen, Tao Zheng, Gang Sun

**Affiliations:** 1 Department of Cardiology, The First Affiliated Hospital of Guizhou University of Traditional Chinese Medicine, # 83 Zhongshan Road, Gui Yang City, Gui Zhou Province 550001 China; 2 Department of Preventive Medicine, Guizhou University of Traditional Chinese Medicine, 4 Dongjing Road, Gui Yang City, Gui Zhou Province 550001 China

**Keywords:** Lipid accumulation product (LAP), Cardiovascular disease (CVD), China health and retirement longitudinal study (CHARLS), U-shaped relationship

## Abstract

**Background:**

Cardiovascular disease (CVD) poses a major health challenge in China. Lipid accumulation product (LAP), reflecting body fat distribution, has been associated with CVD risk. However, evidence regarding the relationship between LAP and CVD among Chinese populations remains limited.

**Methods:**

This study utilized data from the China Health and Retirement Longitudinal Study, a large national cohort study. A total of 4,481 participants aged ≥ 45 years without CVD at baseline were included, with a 9-year follow-up. Cox regression analyses were performed to examine the association between LAP and CVD incidence. Subgroup and sensitivity analyses were also conducted.

**Results:**

LAP was associated with CVD risk in a U-shaped manner, with higher risks observed at both low and high LAP levels (inflection point = 2.7). For LAP ≤ 2.7, the hazard ratio (HR) for CVD was 0.62 (95% CI: 0.40, 0.96, *P* = 0.0309), indicating a 38% decreased risk with increasing LAP. For LAP > 2.7, the HR was 1.22 (95% CI: 1.04, 1.44, *P* = 0.0153), suggesting a 22% increased risk.

**Conclusion:**

This study revealed a U-shaped nonlinear relationship between LAP and CVD risk in Chinese adults, with risks elevated at both low and high LAP levels. The findings suggest LAP could serve as an effective predictor for CVD among the Chinese population. Further studies are warranted to validate the results.

**Supplementary Information:**

The online version contains supplementary material available at 10.1186/s12889-025-25096-8.

## Background

Cardiovascular disease (CVD) has become a growing concern in China in recent years [1]. According to epidemiological data, approximately 330 million people in China are affected by CVD, with 290 million Chinese, or 21.6% of the population, diagnosed with the disease [2–4]. Recognized risk factors include hypertension, obesity, hyperlipidemia, diabetes mellitus, unhealthy lifestyle, gender, and age [5–7]. The lipid accumulation product (LAP) is an index that combines waist circumference (WC) and serum triglycerides (TG) to reflect body fat accumulation, particularly visceral fat [8]. Research has indicated a positive correlation between LAP and the risks of metabolic syndrome, cardiovascular disease, and all-cause mortality [9–12].

Numerous studies have demonstrated a substantial correlation between LAP and the occurrence of CVD [13–15]. It has been suggested that LAP may be a more effective predictor of CVD risk compared to other commonly used metrics such as BMI and WC [16]. However, it is worth noting that LAP only explains a portion of the variance in CVD risk, suggesting that other factors may also play a role [17–20]. In addition, most existing studies have utilized cross-sectional designs, and there is limited evidence specifically related to Chinese populations.

The purpose of this study is to assess the correlation between LAP and CVD risk based on data from the China Health and Aging Longitudinal Study (CHARLS) cohort. This cohort is a comprehensive and representative multicenter study that covers the socio-economic and health conditions of China’s middle-aged and elderly population. As such, it provides a strong foundation for cardiovascular biomarker research. The study, which utilizes the CHARLS cohort, aims to enhance our understanding of the role of LAP in predicting CVD risk among China’s middle-aged and elderly population.

## Methods

### Data source

The China Health and Retirement Longitudinal Study (CHARLS) is a comprehensive longitudinal research initiative led by the National Development Research Institute at Peking University. Launched in 2008, it includes residents aged 45 and older from 450 villages and neighborhoods in 150 counties and districts in 28 provinces in China [21]. The data, primarily derived from face-to-face interviews, biomarker measurements, and geographic information, cover a wide range of aspects, including the health, socioeconomic status, family relationships, and living conditions of the respondents [21]. The biomarker data provide insights into vital biological parameters such as physical fitness and physiological functioning, while the geographic information reflects the respondents’ living environment. These data, collected using rigorous scientific methods and sampling procedures, accurately represent the health status and quality of life of China’s middle-aged and elderly population. To ensure the accuracy of the survey results, all data collection adhered strictly to internationally recognized quality control standards.

### Study population

**Fig. 1:**
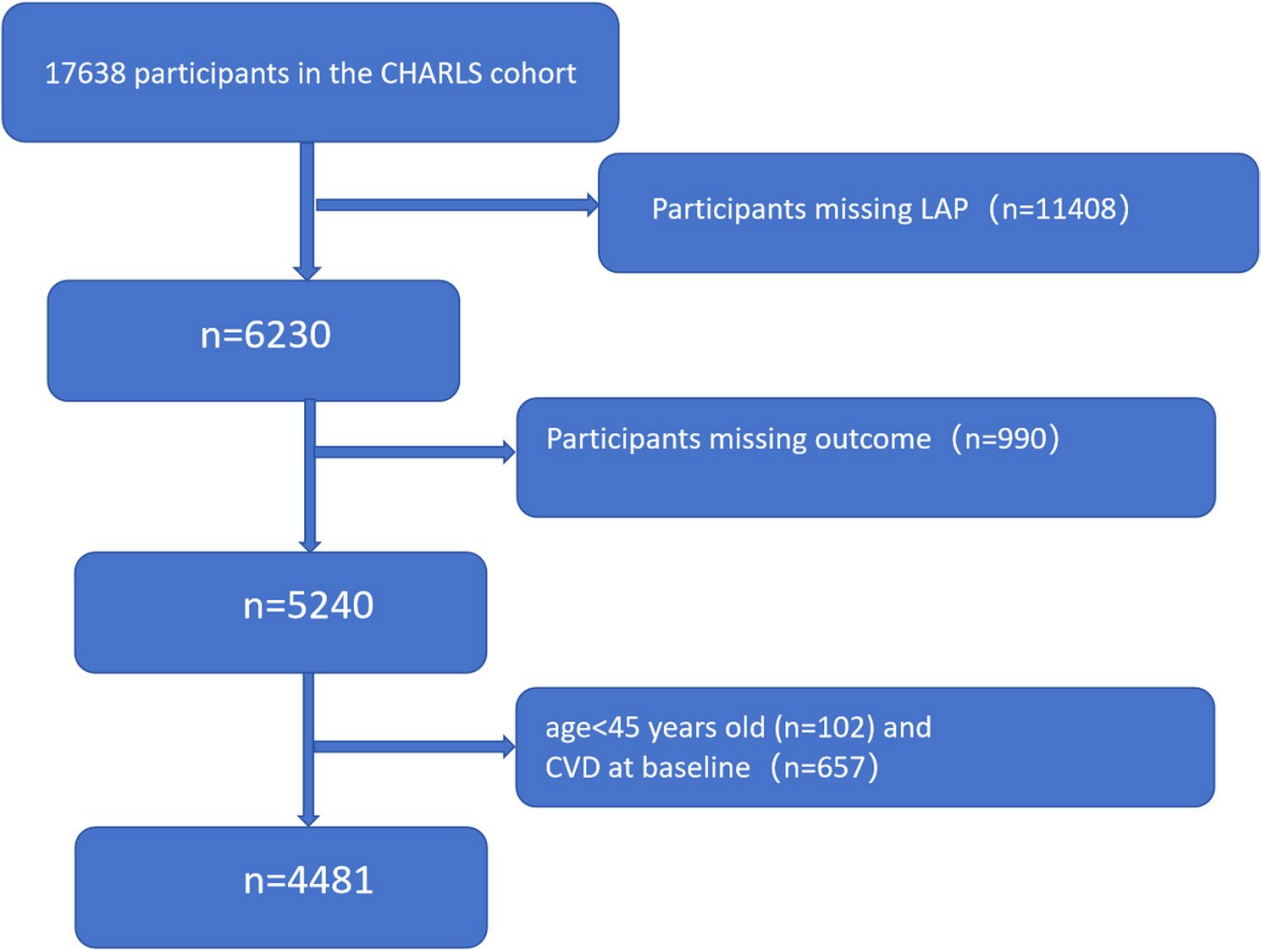
Shows the flow chart of the study procedures, illustrating the process and rationale for selecting patients for final data analysis

The CHARLS cohort includes five waves of survey data (2011, 2013, 2015, 2018, 2020), with 2011 data serving as the baseline and subsequent years as follow-up data. This nationwide survey focuses on a representative sample of Chinese residents aged 45 and older. The baseline survey uses a multistage stratified sampling design proportional to size, covering 150 counties or districts and 450 villages or townships in 28 provinces. The 2011 baseline data included 17,638 individuals. After excluding 11,408 participants with missing LAP data, 6,230 individuals remained. Further exclusions were made for those with missing outcome variables (990), leaving 5,240 participants. Finally, after excluding 102 participants younger than 45 years and 657 patients diagnosed CVD in 2011, 4,481 cases remained for final data analysis (see Fig. 1 for details). All participants provided informed consent prior to enrollment and agreed to participate in the baseline and follow-up examinations. Therefore, all examinations, including blood sampling and various questionnaires, were performed with the informed consent of the participants. The study was approved by the Biomedical Ethics Committee of Peking University (IRB00001052-11015) and adhered to the principles of Strengthening the Reporting of Observational Studies in Epidemiology (STROBE) [22].

### Exposure variables

This study employed the LAP as the exposure variable. LAP is determined by using two anthropometric measurements: waist circumference (WC) and triglyceride levels (TG). It is important to note that the LAP formula differs based on gender. Specifically, for males, LAP is calculated as (WC(cm) − 65) * TG(mmol/L), while for females, it is (WC(cm) − 58) * TG(mmol/L) [8].

### Outcome variables

The study focused on the incidence of heart disease as the outcome variable. According to the CHARLS official user manual, heart disease includes conditions such as myocardial infarction, coronary heart disease, angina pectoris, heart failure, and other heart diseases. The outcome was recorded as a dichotomous variable, with 0 indicating ‘no’ and 1 indicating ‘yes’. These variables were derived from longitudinal follow-up data spanning 2011–2020. The follow-up time was calculated differently depending on whether the outcome occurred or not, or if the data was right-censored. Specifically, if the outcome occurred, the follow-up time was calculated as the difference between the year of outcome occurrence and the baseline year (2011). If the outcome did not occur, the follow-up time was calculated as the difference between the end of the follow-up year (2020) and the baseline year (2011). For right-censored data in this study, the follow-up time was calculated as the difference between the year of the last follow-up and the baseline year (2011).

### Covariates

All covariates, including sociodemographic, comorbidity, treatment-related, and lifestyle data, were collected in the baseline year of 2011 for this study. The sociodemographic data collected included age, educational level, residence, marital status, sex, and insurance. To categorize educational level, we used three categories: elementary school and below, middle/high school/technical school, or college and above. Residence was classified as either rural or urban. Marital status was divided into two categories: single (which includes divorced, living alone, widowed, separated or unmarried) or married. Sex was recorded as either male or female. Insurance was categorized as either New Cooperative Medical Scheme, Rural Cooperative Medical Scheme, or other. The comorbidity data comprises hypertension, dyslipidemia, dysglycemia, stroke, and renal disease. The treatment data comprises lipid-lowering treatment (TCM, Western medicine, or other), antihypertensive treatment (TCM, Western medicine, or other), diabetes treatment (TCM, Western medicine, or other), and stroke-specific treatment (TCM, Western medicine, or other). The lifestyle data provided information on smoking status (smoking, quit smoking, or nonsmoking), alcohol status (drinking, nondrinking, or quit drinking), and physical activity level (inactive, minimally active, moderately active, or vigorously active).

### Data analysis

#### For total population

Before conducting data analysis, missing data was identified and treated appropriately. All indicators had less than 2% missing data, except for physical activity, which had a higher percentage of missing data. Please refer to Supplemental Table 1 for more details. Therefore, missing data for physical activity was treated as a separate category in subsequent analyses. An association between LAP and the incidence of heart disease was observed. To account for the skewed distribution of LAP, the data underwent a natural logarithm transformation. The Ln(LAP) was then categorized into quartiles and the variability in each baseline profile across Ln(LAP) subgroups was assessed. To mitigate potential selection bias due to the high exclusion rate, we employed weighting adjustments provided by CHARLS. Specifically, we used the weight adjustments for biomarker data that account for household and individual-level nonresponse bias. These weighting adjustments are based on the multistage stratified probability-proportional-to-size sampling method employed in the CHARLS cohort, with adjustment factors calculated using response probability models. The adjustment process considered demographic characteristics (age, gender, education level, residence), socioeconomic status, and health conditions, assigning specific weights to each participant to ensure the analyzed sample represents the target population. We also compared the distribution of key variables before and after weighting to assess the effectiveness of the adjustments. Additionally, we have added Supplementary Table 2, which provides a detailed comparison of baseline characteristics between the included (*n* = 4,481) and excluded (*n* = 13,157) populations. Consequently, the study assessed between-group differences in continuous variables using a weighted linear regression model, and between-group differences in categorical variables using a weighted chi-square test. Additionally, a weighted Cox regression model was used to examine the association between LAP and the risk of patients developing heart disease. Three models were constructed: an unadjusted model, a model adjusted for sociodemographic factors only, and a fully adjusted model (adjusted for all variables presented in Table 1). The purpose of these models was to observe the trends in hazard ratio (HR) under different adjustment strategies. Non-linear associations between LAP and CVD risk were assessed using restricted cubic splines with knots positioned at the 5th, 35th, 65th, and 95th percentiles. To optimize knot selection, we compared AIC values across different knot numbers (3, 4, and 5 knots), confirming the 4-knot model as providing the best fit (see Supplemental Table S7). The knot selection was validated through model comparison using the Akaike Information Criterion (AIC), as detailed in Supplemental Table S7.

**Table 1:** Baseline characteristics description of participants in the charls cohort

Ln(LAP)	Q1(−0.51-2.83)	Q2(2.83–3.31)	Q3(3.31–3.81)	Q4(3.81–6.67)	*P* value
Socio-demographic indicators					
Age, mean ± sd, year	60.26 ± 9.59	59.39 ± 9.52	59.60 ± 9.42	59.19 ± 9.25	< 0.001
BMI, mean ± sd, kg/m^2^	20.83 ± 2.62	23.16 ± 3.14	25.05 ± 5.13	27.05 ± 7.17	< 0.0001
Education level %					0.0124
Elementary school and below	67.59	64.85	64.63	64.56	
Middle school, high school, vocational school	31	33.4	34.09	32.61	
College and above	1.41	1.76	1.28	2.84	
Residence %					< 0.0001
Rural	91.42	86.5	80.78	80.68	
Urban	8.58	13.5	19.22	19.32	
Marital status %					0.071
Single (widowed, divorced, separated, never married)	17.58	19.14	18.4	15.73	
Married	82.42	80.86	81.6	84.27	
Sex %					< 0.0001
Male	68.02	47.39	42.26	35.71	
Female	31.98	52.61	57.74	64.29	
Insurance %					< 0.0001
No insurance	5.53	4.33	5.48	7.01	
New Cooperative Medical Scheme	76.56	73.17	70.95	60.65	
Rural Cooperative Medical Scheme	2.26	1.25	1.68	1.63	
Other	15.65	21.25	21.89	30.71	
Outcome-related indicators					
Heart disease (such as myocardial infarction, coronary artery disease, angina, congestive heart failure, and other cardiac conditions) %					0.0016
No	85.32	81.4	81.61	79.17	
Yes	14.68	18.6	18.39	20.83	
Followup time, mean ± sd, year	7.25 ± 2.74	7.38 ± 2.60	7.19 ± 2.70	7.27 ± 2.62	7.25 ± 2.74
Comorbidity					
Hypertension at baseline					< 0.0001
No	82.47	77.78	70.8	62.16	
Yes	17.53	22.22	29.2	37.84	
Dyslipidemia at baseline					< 0.0001
No	94.09	91.82	88.79	80.43	
Yes	5.91	8.18	11.21	19.57	
Dysglycemia at baseline					< 0.0001
No	97.33	94.8	92.83	88.8	
Yes	2.67	5.2	7.17	11.2	
Nephropathy at baseline					0.0709
No	93.05	93	94.9	94.36	
Yes	6.95	7	5.1	5.64	
Stroke at baseline					< 0.0001
No	99.03	96.15	98.44	96.77	
Yes	0.97	3.85	1.56	3.23	
Treatment for Comorbidity					
Use of lipid-lowering drugs % (Traditional Chinese Medicine + Western Medicine + Other)					< 0.0001
No	96.83	94.87	94.36	87.09	
Yes	3.17	5.13	5.64	12.91	
Use of antihypertensive drugs (Traditional Chinese Medicine + Western Medicine + Other)					< 0.0001
No	86.33	81.38	77.45	68.05	
Yes	13.67	18.62	22.55	31.95	
Use of antidiabetic drugs (Traditional Chinese Medicine + Western Medicine + Other)					< 0.0001
No	98.51	96.38	94.78	90.97	
Yes	1.49	3.62	5.22	9.03	
Treatment for stroke(Traditional Chinese Medicine + Western Medicine + Other)					0.0229
No	99.24	97.94	98.81	98.67	
Yes	0.76	2.06	1.19	1.33	
Lifestyle indicators					
Smoking status					< 0.0001
Non-smoker	42.02	60.58	64.39	68.45	
Current smoker	55.41	34.8	32.03	28.59	
Quit smoking	2.57	4.63	3.58	2.96	
Drinking status					< 0.0001
No	58.16	67.78	65.88	75.06	
Yes	9.65	7.86	7.31	5.81	
Quit drinking	32.19	24.36	26.81	19.12	
Physical activity %					0.1444
Inactive	13.18	10.33	12.38	11.43	
Minimally Active	28.09	34.35	30.65	30.54	
Moderately Active	37.56	31.15	32.13	35.93	
Vigorously Active	21.17	24.17	24.84	22.09	

*P* value for continuous variables was calculated by weighted linear regression model

*P* value for categorical variables was calculated by weighted chi-square test

In this study, we weighted the biomarker data to account for bias at both the household and individual levels due to nonresponse. The adjustment calculated aimed to correct for varying participation probabilities across population subsets

Segmented linear regression was employed to identify potential changepoints and quantify effect magnitudes across LAP strata. Additionally, due to the limitations of the Cox model in handling nonlinear associations, a restricted cubic spline was used for smoothed curve fitting. A recursive algorithm was utilized to identify the inflection point where nonlinear associations were detected and 95%CI was calculated by resample methods. Subsequently, a two-piecewise linear model was developed on both sides of this point to accommodate these nonlinear associations.

#### For subgroup analysis

In order to acknowledge the importance of gender and age in CVD-related research and large-scale population studies, the analysis in this study was stratified by gender (male/female) and age (over 65 years). The purpose was to evaluate whether the correlation between Ln(LAP) and CVD incidence risk differs among various subgroups.

#### Sensitivity analysis

1. The study conducted a sensitivity analysis by dividing Ln(LAP) into subgroups and performing a trend test to observe the consistency of the results when Ln(LAP) was treated as a continuous or categorical variable.
2. The study conducted sensitivity analyses and presented supplementary results that were both adjusted and unadjusted for physical activity.
3. Given that the pathogenesis of CVD is a long-term process. We excluded participants who were diagnosed with CVD within 3 years after 2011, and reanalyzed the linear and nonlinear associations. Statistical significance was determined by a *p*-value of less than 0.05 (two-tailed). All analyses were performed using the R statistical software package (The R Foundation).
4. To evaluate extreme value influence, we conducted sensitivity analyses using 1 st and 99th percentile Winsorization.

## Results

### Baseline characteristics of participants in the CHARLS cohort

LAP demonstrated marked positive skewness (mean 40.9 ± 37.3), with log-transformation yielding approximate normality (Supplemental Figs. 1 and 2). The restricted cubic spline model with 4 knots demonstrated superior fit compared to alternative specifications, with AIC values of 7210.7 for the 4-knot model versus 7224.3 and 7218.1 for 3-knot and 5-knot models respectively (Supplemental Table S7). Table 1 presents the baseline characteristics of participants in the Charls Cohort, stratified by quartiles of Ln(LAP). A clear trend is observed across the quartiles for several variables. For instance, the mean age of participants ranges from 60.26 years in the first quartile to 59.19 years in the fourth quartile, with a significant *p*-value of < 0.001. Similarly, BMI increases significantly from 20.83 kg/m2 in the first quartile to 27.05 kg/m2 in the fourth quartile. The percentage of participants with higher education increases from 1.41% in the first quartile to 2.84% in the fourth quartile. The prevalence of heart disease also shows an upward trend from 14.68% in the first quartile to 20.83% in the fourth quartile. The use of lipid-lowering drugs, antihypertensive drugs, and antidiabetic drugs also increases across the quartiles, with significant *p*-values of < 0.0001.

### Results of the weighted univariate and multivariate Cox regression models

Table 2 presents the results of weighted univariate and multivariable Cox regression models examining the association between the natural logarithm of the lipid accumulation product (Ln(LAP)) and the risk of cardiovascular diseases. In the non-adjusted model, a unit increase in Ln(LAP) was associated with a 21.4% increase in the hazard of cardiovascular diseases (Hazard Ratio (HR) = 1.214, 95% Confidence Interval (CI) = 1.213 to 1.215, *p* < 0.0001). This association remained significant but attenuated after adjusting for demographic factors (age, sex, insurance, marital status, residence, education level) in Model I (HR = 1.142, 95% CI = 1.141 to 1.143, *p* < 0.0001), and further attenuated after additional adjustment for clinical and lifestyle factors in Model II (HR = 1.093, 95% CI = 1.092 to 1.094, *p* < 0.0001). When Ln(LAP) was categorized into quartiles, a clear dose-response relationship was observed. Compared to the first quartile (Q1), the hazard of cardiovascular diseases was 24.4%, 26.6%, and 41.8% higher in the second (Q2), third (Q3), and fourth (Q4) quartiles, respectively, in the non-adjusted model. This dose-response relationship remained significant but attenuated in the adjusted models.

**Table 2:** The association between Ln(LAP) and risk of cardiovascular diseases: weighted univariate and multivariable Cox regression models

Exposure	Non-adjusted HR, 95%CI, *P* value	Adjust I HR, 95%CI, *P* value	Adjust II HR, 95%CI, *P* value
Ln(LAP)	1.214 (1.213, 1.215) < 0.0001	1.142 (1.141, 1.143) < 0.0001	1.093 (1.092, 1.094) < 0.0001
Q1	1.0	1.0	1.0
Q2	1.244 (1.243, 1.245) < 0.001	1.150 (1.149, 1.151) < 0.001	1.121 (1.120, 1.123) < 0.001
Q3	1.266 (1.264, 1.267) < 0.001	1.149 (1.147, 1.150) < 0.001	1.124 (1.1233, 1.1256) < 0.001
Q4	1.418 (1.417, 1.420) < 0.001	1.168 (1.167, 1.170) < 0.001	1.099 (1.098, 1.101) < 0.001
P for trend	1.108 (1.107, 1.109) < 0.001	1.043 (1.042, 1.044) < 0.001	1.024 (1.023, 1.025) < 0.001

*HR* Hazard ratio, *CI* confidence interval

Non-adjusted model adjust for: None

Adjust I model adjust for: Age; Sex; Insurance; Marital status; residence; education level

Adjust II model adjust for: Age, BMI, Education level, Residence, Marital status, Sex, Insurance, Use of lipid-lowering drugs, Use of antihypertensive drugs, Use of antidiabetic drugs, Treatment for stroke, Smoking status, Drinking status, Physical activity, Hypertension at baseline, Dyslipidemia at baseline, Dysglycemia at baseline, Nephropathy at baseline, stroke at baseline

These findings suggest that higher levels of LAP are independently associated with an increased risk of cardiovascular diseases.

### The results of the nonlinear association between the LAP and CVD incidence

**Fig. 2:**
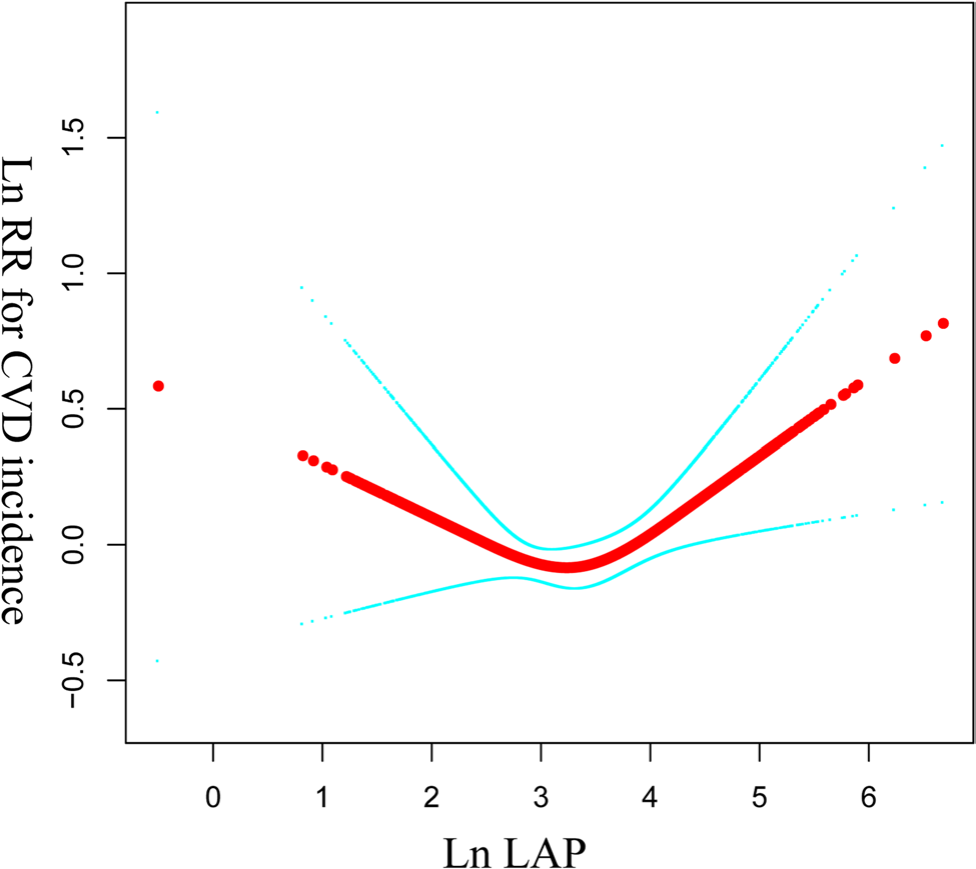
Non-linear Association between Ln(LAP) and LRR for CVD Incidence. The scatter plot illustrates the relationship between the natural logarithm of LAP (Ln(LAP)) and the log relative risk (LRR) for cardiovascular disease (CVD) incidence. The solid red line represents the fitted curve adjusted for all covariates in Model II (Age, BMI, Education level, Residence, Marital status, Sex, Insurance, Use of lipid-lowering drugs, Use of antihypertensive drugs, Use of antidiabetic drugs, Treatment for stroke, Smoking status, Drinking status, Physical activity, Hypertension at baseline, Dyslipidemia at baseline, Dysglycemia at baseline, Nephropathy at baseline, stroke at baseline). The analysis used restricted cubic splines with knots at the 5th, 35th, 65th, and 95th percentiles. The blue dashed lines indicate the 95% confidence interval for this fitted curve. The x-axis represents Ln(LAP), ranging from 0 to 6, while the y-axis represents LRR for CVD incidence, ranging from -0.1 to 0.1

Figure 2 shows that the relationship between Ln(LAP) and the incidence of CVD is U-shaped. Table 3 presents the results of a two-piecewise linear regression model examining the non-linear association between the natural logarithm of the lipid accumulation product (Ln(LAP)) and the risk of cardiovascular diseases. The inflection point of Ln(LAP) was found to be 2.7 (95%CI: 2.4–2.9). For values of Ln(LAP) less than or equal to this inflection point, each unit increase in Ln(LAP) was associated with a 38% decrease in the hazard of cardiovascular diseases (Hazard Ratio (HR) = 0.62, 95% Confidence Interval (CI) = 0.40 to 0.96, *p* = 0.0309). This suggests that at lower levels of LAP is associated with increases in LAP. For values of Ln(LAP) greater than the inflection point, each unit increase in Ln(LAP) was associated with a 22% increase in the hazard of cardiovascular diseases (HR = 1.22, 95% CI = 1.04 to 1.44, *p* = 0.0153). The *p*-value for the log-likelihood ratio test was 0.014, suggesting that the two-piecewise linear model provides a significantly better fit to the data than a Cox regression model.

**Table 3:** The results of the non-linear association are presented using a two-piecewise linear regression model

Ln(LAP)	HR, 95%CI, *P* value
Inflection point (95%CI)	2.7 (2.4, 2.9)
≤ 2.7 (Ln(LAP))	0.62 (0.40, 0.96) 0.0309
> 2.7 (Ln(LAP))	1.22 (1.04, 1.44) 0.0153
*P* for log-likely ratio test	0.014

adjust for: Age, BMI, Education level, Residence, Marital status, Sex, Insurance, Use of lipid-lowering drugs, Use of antihypertensive drugs, Use of antidiabetic drugs, Treatment for stroke, Smoking status, Drinking status, Physical activity, Hypertension at baseline, Dyslipidemia at baseline, Dysglycemia at baseline, Nephropathy at baseline, stroke at baseline

Due to methodological limitations, we are unable to obtain weighted values from the two-piecewise linear model. Therefore, we present the unweighted results

### Subgroup analysis using

**Fig. 3:**
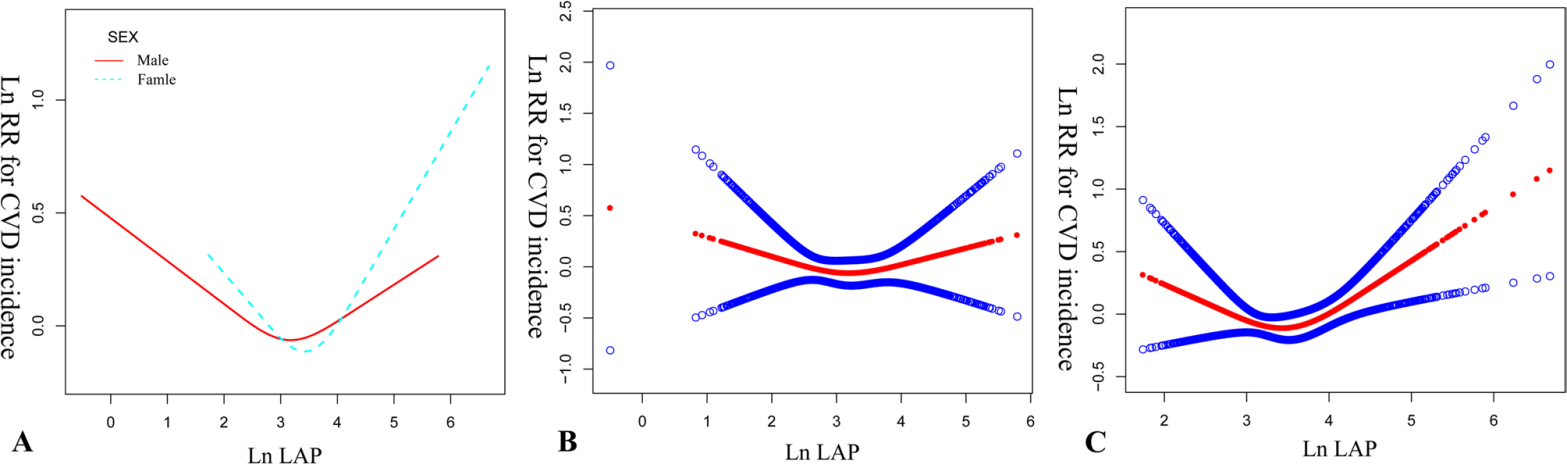
Ln RR for CVD Incidence against Ln(LAP) Stratified by Sex and Age. The figure presents three separate graphs (**A**, **B**, **C**) depicting the natural logarithm of the relative risk (Ln RR) for CVD incidence against the natural logarithm of LAP (Ln(LAP)). Graph **A** represents the data for both sexes with a dashed line for males and a solid line for females. Graph **B** includes data for males, represented by circles connected by a solid line. Graph **C** includes data for females, represented by dots connected by a solid line

This study conducted an exploratory investigation into the U-shaped association between Ln(LAP) and the incidence of CVD, with age and gender as stratification variables. Table 4; Fig. 3 presents the results of the non-linear association between the natural logarithm of LAP (Ln(LAP)) and the incidence of CVD in male and female. The inflection points for males and females are 2.57 (95% CI: 2.34, 2.79) and 2.87 (95% CI: 2.51, 3.06), respectively. For values less than or equal to the inflection point, the hazard ratio (HR) for males is 0.61 (95% CI: 0.34, 1.09, *P* = 0.0951), indicating a non-significant decrease in CVD incidence with increasing Ln(LAP). For females, the HR is 0.41 (95% CI: 0.19, 0.93, *P* = 0.0317), suggesting a significant decrease in CVD incidence with increasing Ln(LAP). For values greater than the inflection point, the HR for males is 1.15 (95% CI: 0.88, 1.50, *P* = 0.2962), indicating a non-significant increase in CVD incidence with increasing Ln(LAP). For females, the HR is 1.32 (95% CI: 1.06, 1.64, *P* = 0.0124), suggesting a significant increase in CVD incidence with increasing Ln(LAP). The *P* values for the log-likelihood ratio tests are 0.094 for males and 0.018 for females, indicating a significant non-linear association between Ln(LAP) and CVD incidence in females but not in males.

**Table 4:** The results of the non-linear association between Ln(LAP) and CVD incidence stratified by sex

Ln(LAP)	Sex = male HR, 95%CI, *P* value	Sex = female HR, 95%CI, *P* value
Inflection point (95%CI)	2.57 (2.34, 2.79)	2.87 (2.51, 3.06)
≤ inflection point	0.61 (0.34, 1.09) 0.0951	0.41 (0.19, 0.93) 0.0317
> inflection point	1.15 (0.88, 1.50) 0.2962	1.32 (1.06, 1.64) 0.0124
*P* for log-likely ratio test	0.094	0.018

Adjust for: Age, BMI, Education level, Residence, Marital status, Insurance, Use of lipid-lowering drugs, Use of antihypertensive drugs, Use of antidiabetic drugs, Treatment for stroke, Smoking status, Drinking status, Physical activity, Hypertension at baseline, Dyslipidemia at baseline, Dysglycemia at baseline, Nephropathy at baseline, stroke at baseline. The reason why the gender variable has not been adjusted is because it is being treated as a stratification variable

**Fig. 4:**
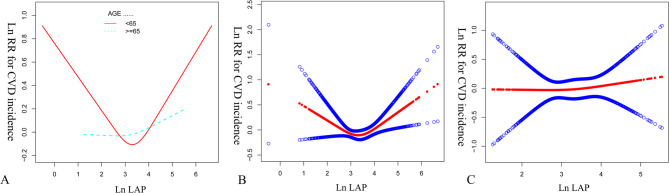
Ln RR for CVD Incidence against Ln(LAP) Stratified by Age. The figure presents three separate graphs (**A**, **B**, **C**) depicting the natural logarithm of the relative risk (Ln RR) for cardiovascular disease (CVD) incidence against the natural logarithm of LAP (Ln(LAP)). Graph **A** represents the total population with two lines indicating different age groups: a solid red line for individuals aged over 65 and a dashed blue line for those aged 65 or under. Graph **B** includes data for individuals aged 65 or under, represented by individual data points plotted along with a smooth curve. Graph **C** includes data for individuals aged over 65, represented by individual data points plotted along with a smooth curve

Table 5; Fig. 4 presents the results of the non-linear association between LAP and the incidence of CVD, stratified by age. The inflection points for individuals aged less than 65 and those aged 65 or older are 2.68 and 2.06, respectively. For values less than or equal to inflection points, the hazard ratio (HR) for individuals aged less than 65 is 0.52 (95% CI: 0.32, 0.84, *P* = 0.0079), indicating a significant decrease in CVD incidence with increasing LAP. For individuals aged 65 or older, the HR is 7.07 (95% CI: 0.12, 414.89, *P* = 0.3465), suggesting a non-significant increase in CVD incidence with increasing LAP. For values greater than inflection points, the HR for individuals aged less than 65 is 1.26 (95% CI: 1.04, 1.51, *P* = 0.0162), indicating a significant increase in CVD incidence with increasing LAP. For individuals aged 65 or older, the HR is 1.00 (95% CI: 0.75, 1.34, *P* = 0.9916), suggesting no significant association between LAP and CVD incidence. The *P* values for the log-likelihood ratio tests are 0.006 for individuals aged less than 65 and 0.274 for those aged 65 or older, indicating a significant non-linear association between LAP and CVD incidence in individuals aged less than 65 but not in those aged 65 or older.

**Table 5:** The results of the non-linear association between LAP and CVD incidence stratified by age

Ln(LAP)	Age < 65 HR, 95%CI, *P* value	Age > = 65 HR, 95%CI, *P* value
Inflection point (95%CI)	2.68 (2.39 to 2.9)	2.06 (2.0 to 2.54)
≤ inflection point	0.52 (0.32, 0.84) 0.0079	7.07 (0.12, 414.89) 0.3465
> inflection point	1.26 (1.04, 1.51) 0.0162	1.00 (0.75, 1.34) 0.9916
*P* for log-likely ratio test	0.006	0.274

Adjust for: Sex, BMI, Education level, Residence, Marital status, Insurance, Use of lipid-lowering drugs, Use of antihypertensive drugs, Use of antidiabetic drugs, Treatment for stroke, Smoking status, Drinking status, Physical activity, Hypertension at baseline, Dyslipidemia at baseline, Dysglycemia at baseline, Nephropathy at baseline, stroke at baseline. The reason why the age variable has not been adjusted is because it is being treated as a stratification variable

### Sensitivity analyses

We conducted a comparative analysis between participants included in the final analysis (*n* = 4,481) and those excluded due to missing key data (*n* = 13,157) shown in supplemental Table 2. Our comparison demonstrates a high degree of similarity in baseline characteristics between the included and excluded groups. No significant differences were observed in key demographic characteristics including age (59.26 ± 9.74 years vs. 59.02 ± 9.82 years; *p* = 0.182) and BMI (23.52 ± 4.00 kg/m² vs. 23.57 ± 3.88 kg/m²; *p* = 0.532). Gender distribution was nearly identical between groups (male: 46.68% vs. 46.32%; *p* = 0.698). Although statistically significant differences were detected in socioeconomic factors such as education level (*p* < 0.001), residence type (*p* = 0.006), marital status (*p* < 0.001), and insurance coverage (*p* < 0.001), the absolute differences were small with all standardized differences below 0.2, indicating these differences were not clinically meaningful. Importantly, the groups showed similar profiles in key health condition indicators, including the prevalence of hypertension (25.39% vs. 24.16%; *p* = 0.082), dyslipidemia (9.32% vs. 9.24%; *p* = 0.864), and diabetes (5.76% vs. 5.68%; *p* = 0.843). Treatment patterns were also comparable between groups, as evidenced by similar usage rates of lipid-lowering medications (5.42% vs. 5.07%; *p* = 0.342) and antidiabetic drugs (4.15% vs. 3.98%; *p* = 0.599). Only baseline stroke prevalence showed a small but statistically significant difference (2.01% vs. 2.51%; *p* = 0.044). Regarding lifestyle characteristics, although smoking status exhibited statistically significant differences (*p* = 0.002), the actual distributions remained highly similar, and physical activity levels showed no significant differences between groups (*p* = 0.269). Supplemental Table 3 presents the changes in the association between LAP and the incidence of CVD when adjusting for physical activity or not. The inflection point for both the original results and the results not adjusted for physical activity is 2.7. For values less than or equal to 2.7 (Ln(LAP)), the hazard ratio (HR) for the original results is 0.62 (95% CI: 0.40, 0.96, *P* = 0.0309), indicating a significant decrease in CVD incidence with increasing LAP. When not adjusted for physical activity, the HR is 0.63 (95% CI: 0.41, 0.98, *P* = 0.0390), suggesting a similar decrease in CVD incidence with increasing LAP. For values greater than 2.7 (Ln(LAP)), the HR for the original results is 1.22 (95% CI: 1.04, 1.44, *P* = 0.0153), indicating a significant increase in CVD incidence with increasing LAP. When not adjusted for physical activity, the HR is 1.22 (95% CI: 1.04, 1.43, *P* = 0.0175), suggesting a similar increase in CVD incidence with increasing LAP. This association appears to be robust to the adjustment for physical activity. The same results can be seen in Supplemental Tables 4 and 5. The results show that even after excluding participants who had CVD within 3 years, the linear and non-linear associations between Ln(LAP) and the risk of CVD incidence do not change significantly. Sensitivity analysis excluding extreme values (1st and 99th percentiles) confirmed the robustness of the U-shaped association, with narrowed confidence intervals and preserved statistical significance of the overall non-linear pattern (*P* < 0.05).

Sensitivity analysis excluding extreme values (1st and 99th percentiles) confirmed the robustness of the U-shaped association (Supplemental Fig. 3), with narrowed confidence intervals and preserved statistical significance of the overall non-linear pattern (*P* < 0.05). To provide additional methodological triangulation, we employed segmented linear regression using Winsorized data, which identified a changepoint at Ln(LAP) = 2.7 (corresponding to LAP ≈ 14.9). The lower segment (< 2.7) demonstrated HR = 0.50 (95% CI: 0.27–0.94, *P* = 0.032), while the upper segment (> 2.7) showed HR = 1.12 (95% CI: 0.94–1.34, *P* = 0.206). The between-segment difference was statistically significant (HR = 2.24, 95% CI: 1.10–4.56, *P* = 0.026), with a log-likelihood ratio test *P*-value of 0.033, confirming the overall non-linear pattern.

## Discussion

This study, utilizing the China Health and Retirement Longitudinal Study (CHARLS) cohort, investigated the association between Lipid Accumulation Product (LAP) and cardiovascular disease (CVD) risk among urban Chinese adults aged over 45. Over nine years of follow-up, we identified a U-shaped relationship, with both high and low LAP levels linked to increased CVD risk, particularly in women and those under 65 years of age. A LAP range of 10–20 (Ln2.3–Ln3) was associated with the lowest risk, offering a potential benchmark for risk stratification.

Previous studies have shown a positive correlation between LAP and the risk of CVD, which is consistent with our findings regarding the association between high LAP and increased CVD risk. For instance, a cohort study of 3042 Greek patients demonstrated a positive association between LAP and the 10-year risk of CVD incidence [13]. Similarly, a cross-sectional survey of 9180 US citizens found that LAP was positively correlated with CVD incidence and was a better indicator than BMI in identifying Americans with CVD [16]. However, our research utilized data from the CHARLS cohort and employed a more advanced algorithm to explore the nonlinear association between LAP and CVD risk, revealing for the first time a U-shaped relationship in the Chinese population, which represents an important complement and extension to previous research.

The potential mechanisms by which elevated LAP may increase CVD risk likely involve multiple pathophysiological pathways. Excessive visceral fat accumulation promotes the release of pro-inflammatory cytokines such as IL-6 and TNF-α, leading to chronic low-grade inflammation [23]. Lipotoxicity resulting from ectopic fat deposition can impair endothelial function through increased oxidative stress and reduced nitric oxide bioavailability [24]. Elevated LAP is strongly associated with insulin resistance, which leads to compensatory hyperinsulinemia that can promote vascular smooth muscle cell proliferation and macrophage activation [25]. Visceral adiposity and elevated triglycerides are associated with a proatherogenic lipid profile characterized by small dense LDL particles, which are more susceptible to oxidation and more readily taken up by arterial wall macrophages [26]. Additionally, elevated LAP is closely linked to metabolic dysfunction-associated steatotic liver disease (MASLD), which may independently contribute to increased cardiovascular risk through hepatic production of inflammatory mediators and procoagulant factors [27].

Our study also reports for the first time in a Chinese population an association between low LAP and high CVD risk. Low LAP often indicates severe malnutrition and wasting disease, with mechanisms for increased CVD risk that differ from those of high LAP but are equally important to understand. Malnutrition can lead to reduced cardiac muscle mass and impaired myocardial function, increasing susceptibility to cardiovascular events [28]. Protein-energy malnutrition may compromise the integrity of the arterial wall by reducing the synthesis of structural proteins and impairing repair mechanisms [29]. Severe nutritional deficiencies can impair the body’s antioxidant defense systems and are associated with immune system dysregulation [30]. Furthermore, low triglyceride levels are frequently accompanied by high HDL-C levels, which may increase CVD risk above certain thresholds, possibly due to dysfunctional properties of HDL particles in certain metabolic states [31].

We found that the U-shaped relationship between LAP and CVD risk was more pronounced in females and in individuals under 65 years of age, reflecting complex physiological and pathological mechanisms. Regarding gender differences, estrogen in women may play an important role. Estrogen regulates lipid metabolism, promoting subcutaneous fat storage while reducing visceral fat accumulation [32], and possesses anti-inflammatory and antioxidant properties [33]. The differences in body fat distribution between men and women are also crucial: women typically have more subcutaneous fat, while men accumulate more visceral fat [34].

Regarding age-related differences, the more pronounced U-shaped relationship observed in populations under 65 years of age may be attributed to multiple factors. Body fat distribution patterns change with age, with increased visceral fat and decreased subcutaneous fat [35]. The ‘obesity paradox’ phenomenon common in elderly populations may disrupt the relationship between LAP and CVD risk, where mild to moderate obesity may have a protective effect in older adults [36]. Comorbidities and medication use are more prevalent in elderly populations, which may act as confounding factors [37]. Additionally, sex hormone levels decrease with age, potentially altering lipid metabolism and cardiovascular protection mechanisms [38].

LAP is a simple indicator that only requires measurements of waist circumference and triglyceride levels, which are easily obtainable in clinical practice. The Fatty Liver Index (FLI) is another widely used metabolic risk assessment tool that combines four parameters: waist circumference, BMI, GGT, and triglycerides [39]. LAP primarily reflects visceral fat accumulation and lipid metabolism abnormalities, is simple to calculate, and is suitable for large-scale screening; while FLI, by incorporating the liver function marker GGT, may better capture risks associated with hepatic steatosis [40]. Studies have shown that FLI is significantly associated with subclinical atherosclerosis and increased risk of cardiovascular events [41]. However, the main advantage of LAP compared to FLI is its simplicity and minimal requirements for basic resources, making it particularly suitable for primary care settings [8].

It is worth noting that LAP is closely associated with metabolic dysfunction-associated steatotic liver disease (MASLD), which may be an important mediating mechanism in the association between LAP and CVD risk. Studies have shown that LAP is an effective predictor of MASLD [42], which is biologically plausible as both components of LAP are directly related to hepatic fat content. Waist circumference reflects visceral fat accumulation, which can release free fatty acids and pro-inflammatory factors directly to the liver via the portal vein [43], while serum triglyceride levels partly reflect the state of hepatic lipid metabolism. MASLD has been widely recognized as an independent risk factor for CVD [44]. ‘Non-alcoholic fatty liver disease’ (NAFLD) has recently been renamed ‘metabolic dysfunction-associated steatotic liver disease’ (MASLD) to more accurately reflect its close relationship with metabolic dysregulation [45]. MASLD may promote CVD development through systemic inflammation, insulin resistance, atherogenic lipid profiles, and prothrombotic states [27, 46]. In the U-shaped relationship between LAP and CVD risk, the role of MASLD may be complex. For high LAP values, the association with increased CVD risk may be partly mediated by MASLD; for low LAP values, advanced liver disease patients may experience liver dysfunction leading to reduced triglyceride synthesis and weight loss [47].

The clinical significance of this study lies in elucidating the U-shaped relationship between LAP and CVD risk in urban Chinese residents, identifying that this relationship is more pronounced in women and individuals under 65 years of age, and suggesting that a LAP range of 10–20 may be associated with the lowest CVD risk, providing reference for future development of LAP standards for the Chinese population. The study is based on the CHARLS cohort, with advantages including large scale, national coverage, prospective design, multi-center approach, and representativeness. Extensive sensitivity analyses were conducted, advanced statistical methods revealed previously unreported U-shaped associations, a wide range of potential confounders were adjusted for, and the nine-year follow-up period allowed comprehensive assessment of the long-term relationship between LAP and CVD risk.

### Limitation

Several limitations should be acknowledged in this study. First, although we conducted comprehensive sensitivity analyses, the observational nature of this study limits causal inferences about the relationship between LAP and CVD risk. Second, LAP measurements were obtained at a single time point, and we could not account for potential changes in LAP levels over the follow-up period. Third, while we adjusted for a comprehensive set of confounders, residual confounding from unmeasured factors cannot be completely excluded. Fourth, the study population was limited to Chinese adults aged 45 years and older, which may limit the generalizability of our findings to younger populations or other ethnic groups. Fifth, CVD events were primarily identified through self-reported diagnoses, which may introduce recall bias, although this was partially mitigated by medical record verification. Finally, despite our extensive sensitivity analyses addressing extreme values, the wide confidence intervals at the distributional extremes indicate some uncertainty in these regions that warrants cautious interpretation.

## Conclusion

Based on this large-scale prospective cohort study, our findings reveal a robust U-shaped nonlinear relationship between LAP levels and cardiovascular disease risk in the Chinese population. The optimal LAP level for minimal CVD risk was identified at approximately 14.9, with both lower and higher levels associated with increased cardiovascular risk. These results suggest that LAP could serve as a valuable biomarker for cardiovascular risk stratification and support the development of personalized prevention strategies. Further validation in diverse populations and investigation of underlying biological mechanisms are warranted.

## Supplementary Information

Supplementary Material 1.

Supplementary Material 2.

## Data Availability

The datasets produced or analyzed during the present study are available in the China Health and Retirement Longitudinal Study repository [http://charls.pku.edu.cn].

## Abbreviations

CVD: Cardiovascular disease
CHARLS: China Health and Retirement Longitudinal Study
LAP: Lipid accumulation product
BMI: Body mass index
HR: Hazard ratio
CI: Confidence interval
TCM: Traditional Chinese Medicine
STROBE: Strengthening the Reporting of Observational Studies in Epidemiology
Ln(LAP): Natural logarithm of the lipid accumulation product
LRR: Log relative risk
