# Supplementary material for: Revealing the U-shaped nonlinear relationship between lipid accumulation product levels and cardiovascular disease risk using the large CHARLS cohort study in China
Source: BMC Public Health. 2025 Nov 14;25:3944. doi: 10.1186/s12889-025-25096-8 (PMC12619476; doi:10.1186/s12889-025-25096-8)

Supplemental table 1: The description of missing status of covariate

| Variables | Without missing | Missing | Missing rate(%) |
| --- | --- | --- | --- |
| Age | 4481 | 0 | 0 |
| BMI | 4417 | 64 | 1.428252622 |
| Education levle | 4478 | 3 | 0.066949342 |
| Residence | 4478 | 3 | 0.066949342 |
| Martial status | 4481 | 0 | 0 |
| Sex | 4481 | 0 | 0 |
| Insurance | 4480 | 1 | 0.022316447 |
| Lipid lowing drugs | 4481 | 0 | 0 |
| Anti hypertension | 4481 | 0 | 0 |
| antidiabetic drugs | 4481 | 0 | 0 |
| Treatment for stroke | 4481 | 0 | 0 |
| hypertension | 4467 | 14 | 0.312430261 |
| Dyslipidemia | 4411 | 70 | 1.562151306 |
| Diabetes | 4449 | 32 | 0.714126311 |
| Stroke | 4476 | 5 | 0.111582236 |
| Renal disease | 4473 | 8 | 0.178531578 |
| Physical activity | 1825 | 2656 | 59.27248382 |
| Smoking status | 4480 | 1 | 0.022316447 |
| Drinking status | 4481 | 0 | 0 |

Supplemental table 2: The description between missing and without missing data

|  | **MISSING** | **WITHOUT MISSING** | **P-value** |
| --- | --- | --- | --- |
| N | 13157 | 4481 | - |
| ****Socio-demographic indicators**** |  |  |  |
| Age, mean±sd, year | 59.02 ± 9.82 | 59.26 ± 9.74 | 0.182 |
| BMI, mean±sd, kg/m2 | 23.57 ± 3.88 | 23.52 ± 4.00 | 0.532 |
| Education level % |  |  | <0.001 |
| Elementary school and below | 8949 (68.02%) | 3155 (70.40%) |  |
| Middle school, high school, vocational school | 3929 (29.86%) | 1276 (28.48%) |  |
| College and above | 279 (2.13%) | 50 (1.11%) |  |
| Residence % |  |  | 0.006 |
| Rural | 11601 (88.17%) | 4023 (89.78%) |  |
| Urban | 1556 (11.83%) | 458 (10.22%) |  |
| Marital status % |  |  | <0.001 |
| Single (widowed, divorced, separated, never married) | 2422 (18.41%) | 718 (16.02%) |  |
| Married | 10735 (81.59%) | 3763 (83.98%) |  |
| Sex % |  |  | 0.698 |
| Male | 6094 (46.32%) | 2092 (46.68%) |  |
| Female | 7063 (53.68%) | 2389 (53.32%) |  |
| Insurance % |  |  | <0.001 |
| No insurance | 796 (6.05%) | 244 (5.44%) |  |
| New Cooperative Medical Scheme | 9917 (75.38%) | 3579 (79.88%) |  |
| Rural Cooperative Medical Scheme | 164 (1.25%) | 67 (1.50%) |  |
| Other | 2280 (17.32%) | 591 (13.18%) |  |
| ****Comorbidity**** |  |  |  |
| Hypertension at baseline |  |  | 0.082 |
| No | 9978 (75.84%) | 3344 (74.61%) |  |
| Yes | 3179 (24.16%) | 1137 (25.39%) |  |
| Dyslipidemia at baseline |  |  | 0.864 |
| No | 11941 (90.76%) | 4064 (90.68%) |  |
| Yes | 1216 (9.24%) | 417 (9.32%) |  |
| Dysglycemia at baseline |  |  | 0.843 |
| No | 12410 (94.32%) | 4223 (94.24%) |  |
| Yes | 747 (5.68%) | 258 (5.76%) |  |
| Nephropathy at baseline |  |  | 0.545 |
| No | 12333 (93.74%) | 4190 (93.50%) |  |
| Yes | 824 (6.26%) | 291 (6.50%) |  |
| Stroke at baseline |  |  | 0.044 |
| No | 12826 (97.49%) | 4391 (97.99%) |  |
| Yes | 331 (2.51%) | 90 (2.01%) |  |
| ****Treatment for Comorbidity**** |  |  |  |
| Use of lipid-lowering drugs % |  |  | 0.342 |
| No | 12490 (94.93%) | 4238 (94.58%) |  |
| Yes | 667 (5.07%) | 243 (5.42%) |  |
| Use of antihypertensive drugs |  |  | 0.066 |
| No | 10656 (80.99%) | 3576 (79.80%) |  |
| Yes | 2501 (19.01%) | 905 (20.20%) |  |
| Use of antidiabetic drugs |  |  | 0.599 |
| No | 12633 (96.02%) | 4295 (95.85%) |  |
| Yes | 524 (3.98%) | 186 (4.15%) |  |
| Treatment for stroke |  |  | 0.310 |
| No | 12938 (98.33%) | 4415 (98.53%) |  |
| Yes | 219 (1.67%) | 66 (1.47%) |  |
| ****Lifestyle indicators**** |  |  |  |
| Smoking status |  |  | 0.002 |
| Non-smoker | 8040 (61.11%) | 2647 (59.06%) |  |
| Current smoker | 4783 (36.35%) | 1686 (37.62%) |  |
| Quit smoking | 334 (2.55%) | 148 (3.32%) |  |
| Drinking status |  |  | 0.233 |
| No | 8785 (66.78%) | 3043 (67.92%) |  |
| Yes | 1062 (8.07%) | 334 (7.46%) |  |
| Quit drinking | 3310 (25.15%) | 1104 (24.62%) |  |
| Physical activity % |  |  | 0.269 |
| Inactive | 578 (11.28%) | 208 (11.64%) |  |
| Minimally Active | 1620 (31.63%) | 548 (30.69%) |  |
| Moderately Active | 1729 (33.75%) | 577 (32.32%) |  |
| Vigorously Active | 1196 (23.35%) | 452 (25.34%) |  |

Supplemental table 3: The changes in the association between LAP and CVD incidence when adjusting for physical activity or not

|  | Original results  HR, 95%CI, P value | Non-Adjusted for physical activity  HR, 95%CI, P value |
| --- | --- | --- |
| Inflection point (95%CI) | 2.7 | 2.7 |
| ≤ 2.7 (Ln Lap) | 0.62 (0.40, 0.96) 0.0309 | 0.63 (0.41, 0.98) 0.0390 |
| > 2.7 (Ln Lap) | 1.22 (1.04, 1.44) 0.0153 | 1.22 (1.04, 1.43) 0.0175 |
| P for log-likely ratio test | 0.014 | 0.018 |

Original results:Age, BMI, Education level, Residence, Marital status, Sex, Insurance, Use of lipid-lowering drugs, Use of antihypertensive drugs, Use of antidiabetic drugs, Treatment for stroke, Smoking status, Drinking status, Physical activity, Hypertension at baseline, Dyslipidemia at baseline, Dysglycemia at baseline, Nephropathy at baseline, stroke at baseline

Non-Adjusted for physical activity: Age, BMI, Education level, Residence, Marital status, Sex, Insurance, Use of lipid-lowering drugs, Use of antihypertensive drugs, Use of antidiabetic drugs, Treatment for stroke, Smoking status, Drinking status, Hypertension at baseline, Dyslipidemia at baseline, Dysglycemia at baseline, Nephropathy at baseline, stroke at baseline

Supplemental table 4: The changes in the association between LAP and CVD incidence when exclude participants who developed CVD within 3 years or not

| Exposure | The results of participants for total population  HR, 95%CI, P value | The results of exclude participants who developed CVD within 3 years  HR, 95%CI, P value |
| --- | --- | --- |
| Ln LAP | 1.093 (1.092, 1.094) <0.0001 | 1.125 (1.124, 1.126) <0.001 |
| Q1 | 1.0 | 1.0 |
| Q2 | 1.121 (1.120, 1.123) <0.001 | 1.148 (1.147, 1.1500) <0.001 |
| Q3 | 1.124 (1.1233, 1.1256) <0.001 | 1.179 (1.178, 1.181) <0.001 |
| Q4 | 1.099 (1.098, 1.101) <0.001 | 1.161 (1.160, 1.163) <0.001 |
| P for trend | 1.024 (1.023, 1.025) <0.001 | 1.148 (1.147, 1.1500) <0.001 |

adjust for: Age, BMI, Education level, Residence, Marital status, Sex, Insurance, Use of lipid-lowering drugs, Use of antihypertensive drugs, Use of antidiabetic drugs, Treatment for stroke, Smoking status, Drinking status, Physical activity, Hypertension at baseline, Dyslipidemia at baseline, Dysglycemia at baseline, Nephropathy at baseline, stroke at baseline

Supplemental table 5: The changes in the non-linear association between LAP and CVD incidence when exclude participants who developed CVD within 3 years or not

|  | The results of participants for total population  HR, 95%CI, P value | The results of exclude participants who developed CVD within 3 years  HR, 95%CI, P value |
| --- | --- | --- |
| Inflection point (95%CI) | 2.7 | 2.7 |
| ≤ 2.7 (Ln Lap) | 0.62 (0.40, 0.96) 0.0309 | 0.61 (0.40, 0.98) 0.0343 |
| > 2.7 (Ln Lap) | 1.22 (1.04, 1.44) 0.0153 | 1.21 (1.07, 1.49) 0.0201 |
| P for log-likely ratio test | 0.014 | 0.020 |

adjust for: Age, BMI, Education level, Residence, Marital status, Sex, Insurance, Use of lipid-lowering drugs, Use of antihypertensive drugs, Use of antidiabetic drugs, Treatment for stroke, Smoking status, Drinking status, Physical activity, Hypertension at baseline, Dyslipidemia at baseline, Dysglycemia at baseline, Nephropathy at baseline, stroke at baseline

Supplemental table 6：Two-piecewise linear mdoel for LAP-CVD Relationship Using 1% and 99% Percentile Winsorization

| Ln(LAP) | HR, 95%CI, P value |
| --- | --- |
| Inflection point (95%CI) | 2.7 (2.4 to 2.9) |
| ≤ 2.7 (Ln Lap) | 0.50 (0.27, 0.94) 0.0316 |
| > 2.7 (Ln Lap) | 1.12 (0.94, 1.34) 0.2064 |
| P for log-likely ratio test | 0.033 |

adjust for: Age, BMI, Education level, Residence, Marital status, Insurance, Use of lipid-lowering drugs, Use of antihypertensive drugs, Use of antidiabetic drugs, Treatment for stroke, Smoking status, Drinking status, Physical activity, Hypertension at baseline, Dyslipidemia at baseline, Dysglycemia at baseline, Nephropathy at baseline, stroke at baseline. The reason why the gender variable has not been adjusted is because it is being treated as a stratification variable.

Table S7: Comparison of AIC values for restricted cubic spline (RCS) models with different numbers of knots in the Cox proportional hazards analysis of LAP and the primary outcome.

| **Knot Number** | **Knot Positions (Percentiles)** | **AIC Value** | **ΔAIC (compared to 4 knots)** |
| --- | --- | --- | --- |
| 3 | 10th, 50th, 90th | 7224.3 | +13.6 |
| 4 | 5th, 35th, 65th, 95th | 7210.7 | 0 (Reference) |
| 5 | 5th, 27.5th, 50th, 72.5th, 95th | 7218.1 | +7.4 |

Knot positions follow Harrell's recommendations. The 4-knot model provides the best fit with the lowest AIC. ΔAIC values >2 indicate substantial support for the 4-knot model over alternatives (Burnham & Anderson, 2002).

Supplemental Figure 1：The distribution trend of LAP value


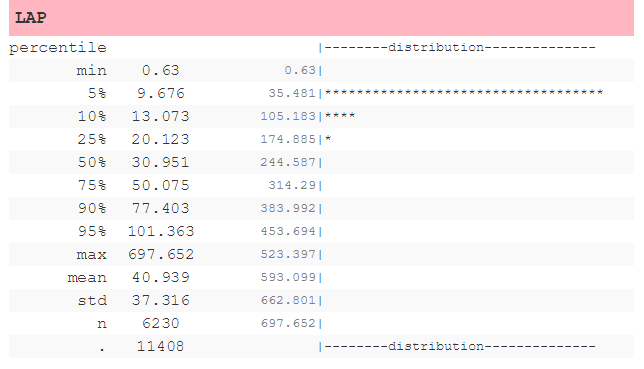


Supplemental Figure 2：The distribution trend of Ln(LAP)


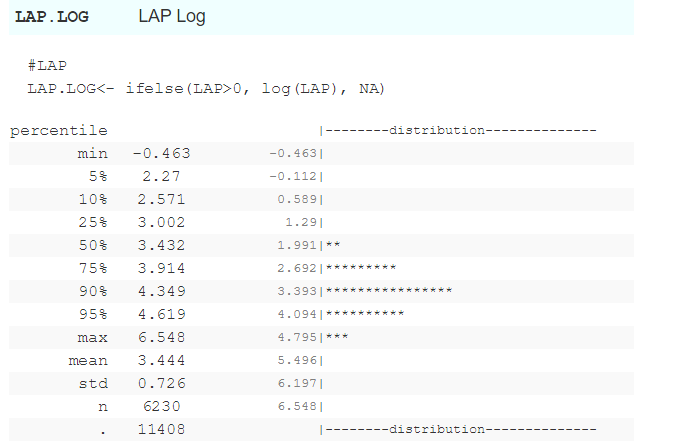


Supplemental Figure 3：Sensitivity Analysis of LAP-CVD Relationship Using 1% and 99% Percentile Winsorization


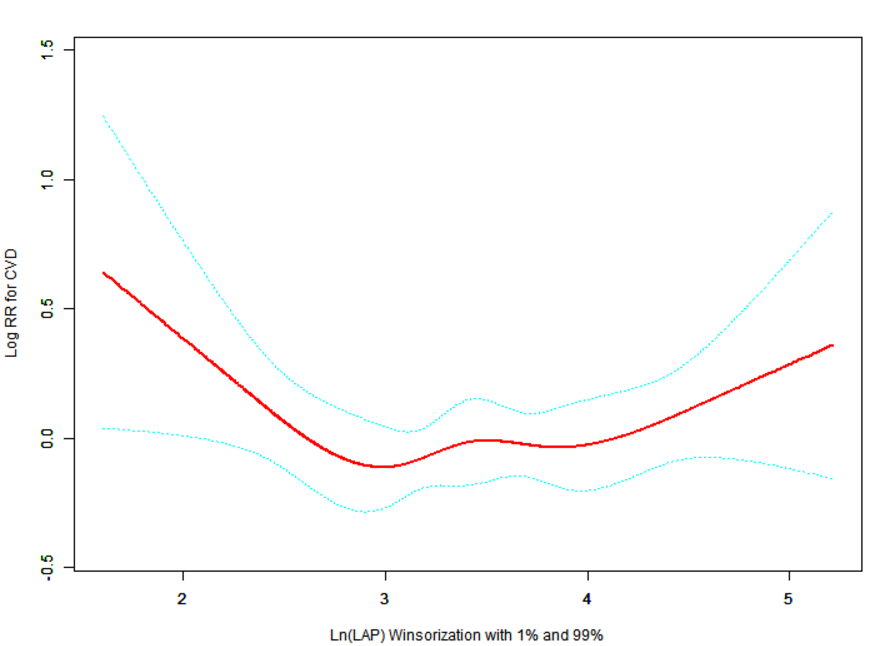

Supplement: Supplementary file 1 — Supplementary Material 1. [file 12889_2025_25096_MOESM1_ESM.docx]
