# Supplementary material for: Revealing the U-shaped nonlinear relationship between lipid accumulation product levels and cardiovascular disease risk using the large CHARLS cohort study in China
Source: BMC Public Health. 2025 Nov 14;25:3944. doi: 10.1186/s12889-025-25096-8 (PMC12619476; doi:10.1186/s12889-025-25096-8)
Supplement: Supplementary file 2 — Supplementary Material 2. [file 12889_2025_25096_MOESM2_ESM.docx]

## **STROBE Checklist**

| **Item** | **Recommendation** | **Page/Section** | **Description** |
| --- | --- | --- | --- |
| ****Title and Abstract**** |  |  |  |
| 1a | Indicate the study's design with a commonly used term in the title or abstract | Page 1 | Title indicates "cohort study" |
| 1b | Provide an informative and balanced summary of what was done and what was found | Page 2 | Abstract contains structured summary with background, methods, results, and conclusion |
| ****Introduction**** |  |  |  |
| 2 | Explain the scientific background and rationale for the investigation | Pages 2-3 | Background section explains the importance of LAP in cardiovascular disease risk prediction |
| 3 | State specific objectives, including any prespecified hypotheses | Page 3 | End of introduction states the study aims to investigate associations between LAP and CVD incidence |
| ****Methods**** |  |  |  |
| 4 | Present key elements of study design early in the paper | Page 3 | Study design described in first paragraph of Methods section |
| 5 | Describe the setting, locations, and relevant dates, including periods of recruitment, exposure, follow-up, and data collection | Page 3 | Setting described including CHARLS cohort, 2011 baseline and 4-year follow-up |
| 6a | Give the eligibility criteria, and the sources and methods of selection of participants. Describe methods of follow-up | Page 3 | Detailed inclusion/exclusion criteria provided and follow-up methods described |
| 6b | For matched studies, give matching criteria and number of exposed and unexposed | N/A | Not a matched study |
| 7 | Clearly define all outcomes, exposures, predictors, potential confounders, and effect modifiers | Pages 3-4 | LAP calculation defined, CVD outcomes described, confounders listed |
| 8 | For each variable of interest, give sources of data and details of methods of assessment | Pages 3-4 | Data sources and assessment methods described for all variables |
| 9 | Describe any efforts to address potential sources of bias | Page 5 | Weighting adjustments for nonresponse bias described |
| 10 | Explain how the study size was arrived at | Page 3 | Sample selection process with numbers at each stage described |
| 11 | Explain how quantitative variables were handled in the analyses | Pages 4-5 | Statistical methods section describes handling of variables |
| 12a | Describe all statistical methods, including those used to control for confounding | Pages 4-5 | Statistical analysis section details methods |
| 12b | Describe any methods used to examine subgroups and interactions | Page 5 | Subgroup analyses by age and sex mentioned |
| 12c | Explain how missing data were addressed | Page 3 | Exclusion criteria include participants with missing data |
| 12d | Explain how loss to follow-up was addressed | Page 5 | Weighting adjustments mentioned to address nonresponse bias |
| 12e | Describe any sensitivity analyses | Page 5 | Sensitivity analyses mentioned |
| ****Results**** |  |  |  |
| 13a | Report numbers of individuals at each stage of study | Page 3, Figure 1 | Flow diagram shows participant selection process |
| 13b | Give reasons for non-participation at each stage | Page 3, Figure 1 | Reasons for exclusion provided |
| 13c | Consider use of a flow diagram | Figure 1 | Flow diagram included |
| 14a | Give characteristics of study participants | Table 1 | Baseline characteristics presented by LAP quartiles |
| 14b | Indicate number of participants with missing data for each variable of interest | Page 3 | Numbers of missing data reported for key variables |
| 14c | Summarize follow-up time | Page 6 | Follow-up period described (4 years) |
| 15 | Report numbers of outcome events or summary measures over time | Pages 6-7, Tables 2-3 | Incidence rates and hazard ratios reported |
| 16a | Give unadjusted estimates and confounder-adjusted estimates | Tables 2-3 | Both unadjusted and adjusted hazard ratios provided |
| 16b | Report category boundaries when continuous variables were categorized | Table 1 | LAP quartile boundaries provided |
| 16c | Consider translating estimates of relative risk into absolute risk | Pages 6-7 | Incidence rates per 1000 person-years reported |
| 17 | Report other analyses done | Pages 7-8, Table 3 | Subgroup analyses by age and sex reported |
| ****Discussion**** |  |  |  |
| 18 | Summarize key results with reference to study objectives | Pages 8-9 | Key findings summarized in first paragraph of Discussion |
| 19 | Discuss limitations of the study, taking into account sources of potential bias or imprecision | Pages 10-11 | Limitations section addresses potential biases |
| 20 | Give a cautious overall interpretation of results considering objectives, limitations, multiplicity of analyses, results from similar studies, and other relevant evidence | Pages 8-10 | Results interpreted in context of existing literature |
| 21 | Discuss the generalizability (external validity) of the study results | Page 10 | Generalizability to Chinese and other populations discussed |
| ****Other Information**** |  |  |  |
| 22 | Give the source of funding and the role of the funders for the present study | Last page | Funding information provided in acknowledgments section |
